# Screening and molecular dynamics simulation of compounds inhibiting MurB enzyme of drug-resistant *Mycobacterium tuberculosis*: An *in-silico* approach

**DOI:** 10.1016/j.sjbs.2023.103730

**Published:** 2023-07-04

**Authors:** Ankit Verma, Vijay Kumar, Bindu Naik, Javed Masood Khan, Pallavi Singh, Per Erik Joakim Saris, Sanjay Gupta

**Affiliations:** a Himalayan School of Biosciences, Swami Rama Himalayan University, Jolly Grant, Dehradun, Uttarakhand, India 248140; b Department of Food Science and Technology, Graphic Era (Deemed to be University), Bell Road, Clement Town, Dehradun 248002, Uttarakhand, India; c Department of Food Science and Nutrition, Faculty of Food and Agricultural Sciences, King Saud University, 2460, Riyadh 11451, Saudi Arabia; d Department of Biotechnology, Graphic Era (Deemed to be University), Bell Road, Clement town, 248002 Dehradun, Uttarakhand, India; e Department of Microbiology, Faculty of Agriculture and Forestry, University of Helsinki, Finland

**Keywords:** Drug resistance, *M. tuberculosis*, Peptidoglycan, MurB, Docking, MD simulation

## Abstract

*Mycobacterium tuberculosis* (MTB) is becoming more and more resistant to drugs and it is a common problem, making current antimicrobials ineffective and highlighting the need for new TB drugs. One of the promising targets for treating MTB is MurB enzymes. This study aimed to identify potential inhibitors of MurB enzymes in *M. tuberculosis*, as drug resistance among MTB is a significant problem. Attempts are being made to conduct a virtual screening of 30,417 compounds, and thirty-two compounds were chosen for further analysis based on their binding conformations. The selected compounds were assessed for their drug-likeness, pharmacokinetics, and physiochemical characteristics, and seven compounds with binding energy lower than flavin (FAD) were identified. Further, molecular dynamics simulation analysis of these seven compounds found that four of them, namely DB12983, DB15688, ZINC084726167, and ZINC254071113 formed stable complexes with the MurB binding site, exhibiting promising inhibitory activity. These compounds have not been mentioned in any other study, indicating their novelty. The study suggests that these four compounds could be promising candidates for treating MTB, but their effectiveness needs to be validated through in vitro and in vivo experiments. Overall, the findings of this study provide new insight into potential drug targets and candidates for combating drug-resistant MTB.

## 1 Introduction

Tuberculosis is a significant global health concern caused by *M. tuberculosis* and is among the top ten deadliest diseases worldwide. It is the primary cause of death from a single infectious agent and is more prevalent than HIV/AIDS (Global TB Report, 2022). According to the WHO, almost 10 million people worldwide fell ill with TB. The report also states that there were 1.5 million TB-related deaths, in 2020, including 214,000 deaths among people with HIV (Global TB Report, 2022). Despite a consistent decrease in TB cases over time, the estimated number of cases increased by 4.5% from 2020 to 2021, indicating a reversal of the previously observed trend (Global TB Report, 2022). TB account for a substantial number of deaths worldwide, particularly among individuals who are HIV-negative. In 2021, the South-East Asia and African regions, along with India, accounted for 36% of these deaths. When considering both HIV-negative and HIV-positive individuals, these regions accounted for 32% of all TB-related deaths. The number of HIV-TB co-infection cases has been alarmingly increasing over the past decade. Although various treatments are available, the evolution of the drug-resistant strain of *M. tuberculosis* highlights the urgent need for new therapeutic approaches to combat multi-drug resistance and HIV-TB co-infection (Konyariková et al., 2020, Kumar et al., 2020). Targeting *Mycobacterium* cell wall synthesis pathways is a promising approach for the development of novel anti-tubercular compounds (Maitra et al., 2019). The sophisticated arrangement of the cell wall structure of *Mycobacterium* species is essential for their survival, pathogenicity, and resistance to various pharmacological therapies (Kumar et al., 2020). The *Mycobacterium* cell wall primarily comprises peptidoglycan (PG), mycolic acid (MA), and arabinogalactan (AG), which together form a complex mAGP. This creates an unusually lipidic and intensely hydrophobic barrier to shield the pathogen from the host’s immune system and traditional antibiotics (Daffé and Marrakchi, 2019). The cross-linked materials that compose its mesh-like configuration are NAG and NAM, repeating glycan units that provide cellular structure and integrity while protecting it from osmotic lysis (Kumar et al., 2020). Several research studies have examined the dynamic nature of PG and its various alterations (Maitra et al., 2019). Peptidoglycan provides structural integrity to bacterial cells and is involved in various vital processes, including cell division, cell shape maintenance, and protection against osmotic stress. Inhibition of peptidoglycan biosynthesis disrupts cell wall formation, ultimately leading to bacterial cell death (Zhang et al., 2012). Therefore, targeting enzymes involved in peptidoglycan biosynthesis represents a promising strategy for developing effective antimicrobial agents against MTB.

Bacterial peptidoglycan production is initiated by a set of murine enzymes called Mur enzymes A-F, which catalyze early cytoplasmic steps. Among these enzymes, MurB plays a vital role in the biosynthesis of bacterial cell walls and is an attractive target for drug development. In MTB, the MurB protein comprises three domains and a secondary element characterized by the α + β combination. Domain I and II are responsible for FAD binding, while domain III interacts with the substrate. Domain, I span from amino acid residues 21 to 81 and 364 to 369, while domain II covers residues 90 to 244. Similarly, domain III comprises residues 25 to 361, with some residues present at the C-terminus (Eniyan et al., 2018).

The catalytic activity of the enzyme-substrate complex of MurB in *Mycobacterium* is facilitated by a monovalent cation and three essential amino acid residues: Arg 176, Glu 361, and Ser 257 play a crucial role in proton transfer during the second reduction step to an enol intermediate (Daffé and Marrakchi, 2019). Arg 176 and Glu 361 are thought to stabilize the enol intermediate through protonation since the oxygen of the enolpyruvylcarboxylate is close to these residues. While the MurB protein interacts with EP-UDP-GlcNAc and FAD through a total of eleven highly conserved residues, seven of these residues, including Asn 71, Tyr 175, Arg 176, Arg 238, Ser 257, His 324, and Glu 361, are essential for the activity (Daffé and Marrakchi, 2019). Blocking these seven amino acid residues can inhibit the catalytic function of the MurB enzyme. Hence, targeting these residues may provide a promising therapeutic approach to combat *M. tuberculosis* infections.

The process of transforming UDP-N-glucosamine into UDP-N-acetylmuramyl involves a succession of enzymes working together, ultimately resulting in the attachment of five peptides to the latter (Kumar et al., 2020). One such enzyme is MurB, which reduces UDP-N-acetylenolpyruvoylglucosamine, a molecule involved in converting UDP-GlcNAc into UDP-MurNAc. After the formation of UDP-MurNAc, MurB adds a PEP enol pyruvyl moiety and reduces the resulting complex with NADPH into a lactose ether moiety (Abrahams and Besra, 2018, Maitra et al., 2019, Kumar et al., 2020). NamH, a UDP-N-acetyl muramic acid hydroxylase, then hydroxylates UDP-MurNAc to produce UDP-N-glycolylmuramic, a substrate that predominates in the Mtb cell wall (Abrahams and Besra, 2018). Enzymes C to F, which are ligases requiring ATP, catalyze the following steps by adding L-alanine to the carboxyl group of UDP-MurNAc, leading to the production of UPD-N-acetylmuramyl-L-alanine (Maitra et al., 2019). These enzymes share functional and structural similarities, including central ATP binding domains, N-terminal domains that bind nucleotides as substrates, and C-terminal domains that bind amino acids as substrates (Rani et al., 2020). Suppression of enzymes involved in initial peptidoglycan biogenesis leads to cell death via cell wall breakdown and lysis (Chen et al., 2018). However, most antibiotics target the final stages of PG production, neglecting the earlier Mur enzyme-catalyzed steps (Hrast et al., 2014). Studies have suggested that the Mur enzymes could be a promising target for developing new drugs (Abrahams and Besra, 2018, Kumar et al., 2011).

Ligand-based computational virtual screening techniques have greatly aided de novo structural characterization, enabling the identification of potential inhibitors for drug repurposing. SBDD is gaining popularity due to its ability to deliver more precise hits against specific targets at a lower cost than the time-consuming process of random screening. The benefits of theoretical prediction and validation of structural modeling, binding effectiveness, and protein–ligand interaction include the reduction of false positives and the eventual enhancement of specificity in identifying potential hits through in vitro validations (Rožman et al., 2017). In particular, drug repurposing increases the likelihood of discovering an inhibitor by employing previously reported drugs or compounds. Recent studies have identified FDA-approved drugs that could counteract Mtb enzymes MurB and MurE (Rani et al., 2020). Additionally, a screening assay was developed to test several furane-based benzenes-derived compounds against MurE and MurF (Eniyan et al., 2016). Despite this, MurB remains one of the least researched targets for finding potential inhibitors.

In this investigation, a Structure-based methodology was employed to conduct a virtual screening of compounds obtained from three repositories, namely ChemSpider, DrugBank*, a*nd the Zinc database. AutoDock Vina was utilized as the docking program, with MurB serving as the protein target. The compounds with the highest binding scores were selected for further analysis. To assess the stability of the protein–ligand interaction, MD simulation (MDS) was performed. MDS is a powerful tool that allows for the study of protein–ligand interactions over a period of time, providing valuable insights into the dynamic behavior of the system. The results obtained from these simulations can help to identify potential inhibitors that may be effective in targeting MurB*.*

## 2 Materials and methods

**Fig. 1:**
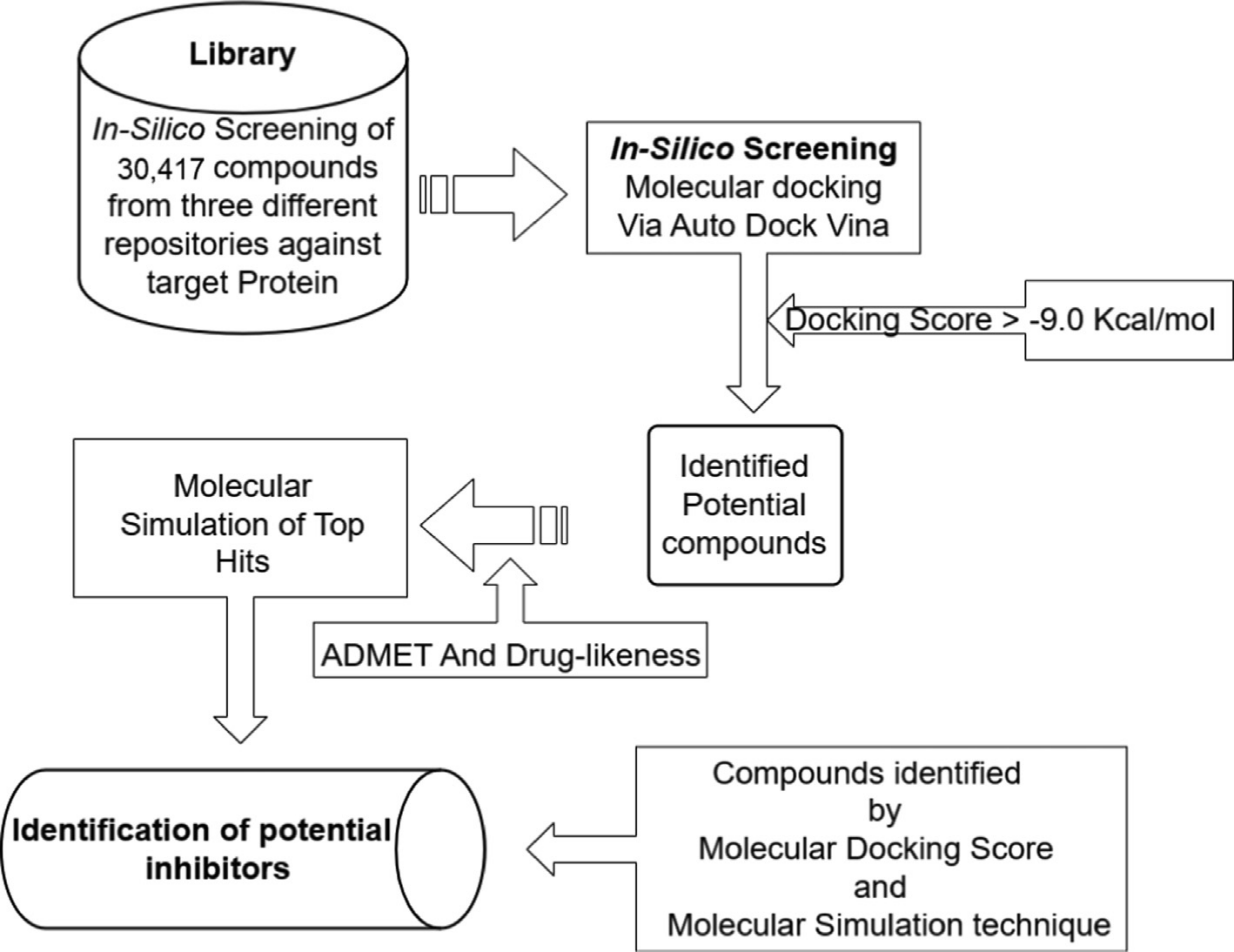
A diagrammatic representation of methodologies employed in the study for the identification of potent inhibiting compounds targeting the MurB enzyme is depicted herein.

Combining molecular docking and the MDS approach, the process used in this study to identify hit compounds was represented in Fig. 1.

### 2.1 Retrieval of compounds from repositories

A set of 30,417 compounds were obtained 10,000 from ChemSpider (Pence and Williams, 2010), 9137 from DrugBank (Wishart et al., 2018), and 11,280 from the Zinc database (Sterling and Irwin, 2015). The selection of these compounds was based on their approval, regulatory authorization in context of proven safety and effectiveness and clinical trials. The compounds used in this study were obtained in SDF format. To enable molecular docking studies, these compounds were converted to PDBQT format using the Open Babe tool, which is a widely used tool for chemical file format conversion (O’Boyle et al., 2011).

### 2.2 Protein structure preparation

**Fig. 2:**
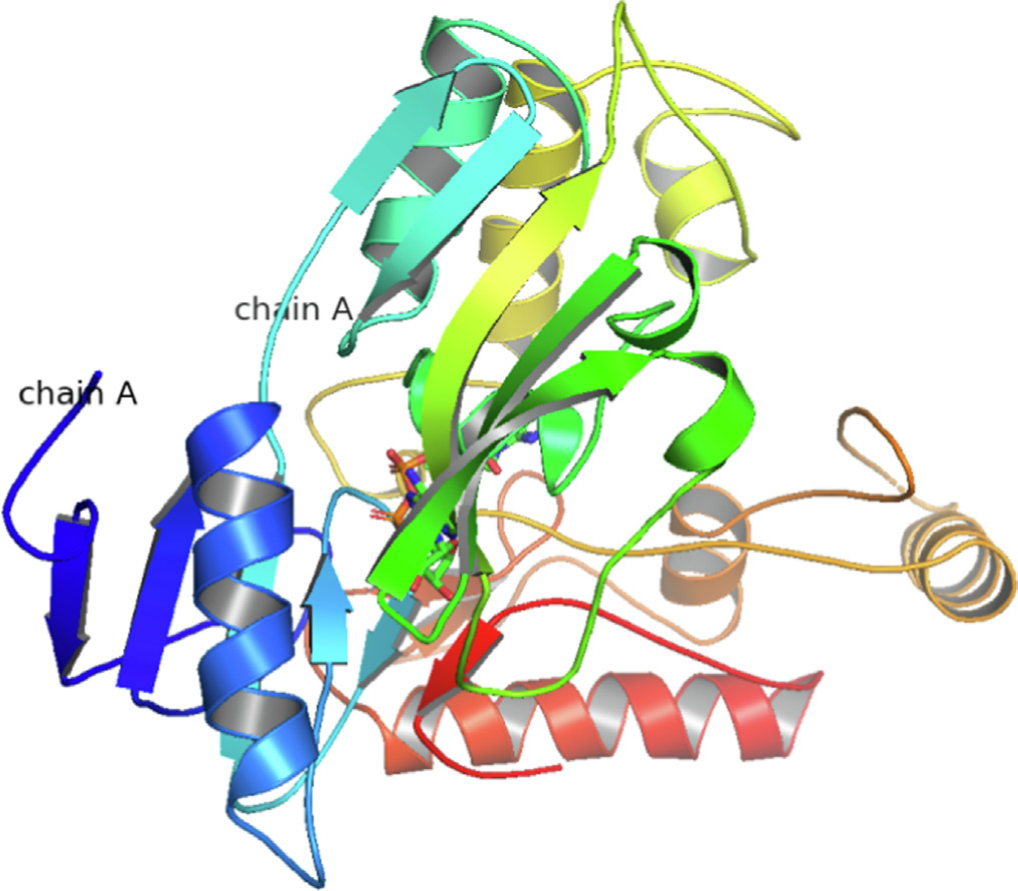
Three-dimensional (3D) structure of MurB (5JZX).

In this study, we obtained the 2.2 Å MurB crystal structure bound to FAD and K+ (PDB:5JZX) (Fig. 2) from the PDB (Eniyan et al., 2018, Eniyan et al., 2020).

The docking studies were performed using the AutoDock tools (version 1.5.6) (Morris et al., 2009). To ensure the reliability and high quality of the protein structure, redundant dimeric units of the crystal structure, which consisted of six MurB molecules, were removed during the pre-processing stage. The MurB protein was protonated with polar hydrogens that had predetermined Kollman charges.

The PDB was converted to a PDBQT file, which carries information about torsional degrees of freedom and partial charges. The MurB protein was fixed but the side chains and the torsional bonds of the ligand were allowed to move freely. Water and hetatms were removed, and the protein structure was repaired to eliminate any overlapping atoms, unwanted loop sections, and asymmetric side chains. This step also ensured that any missing or overlapping atoms and side chains were straightened out. Overall, the preparation of the MurB protein structure for docking was carried out with meticulous attention to detail to ensure that the resulting protein–ligand interactions were stable and reliable.

### 2.3 Grid construction and screening

To identify potential ligands that could interact with MurB and induce the desired therapeutic effect of antibacterial activity, virtual screening was performed. Specifically, a molecular docking approach was used to predict the binding orientation of the ligands to the MurB enzyme. The AutoDock vina script (Trott and Olson, 2010) was utilized to screen a large library of compounds (30,417 in total) against the MurB enzyme of *M. tuberculosis*. The docking was carried out using a blind approach, with a grid map set to 100 for X, Y, and Z dimensions, respectively, and a spacing of 0.5 Å. A Lamarckian genetic method was employed, which incorporated free energy and RMSD values to improve the accuracy of the predictions.

Ten docking runs were carried out, each with a population size of 150 and a maximum of 27 K generations. The maximum generation evaluation was set to 2,500 K. The binding affinity of each compound was calculated in terms of kilocalories per mole (Kcal/mol), and a cut-off of −9.0 Kcal/mol was set as the threshold for screening. The crystal structure of the MurB enzyme and the RMSD values of the docking complexes were taken into account, as well as the inhibition constant (KI).

To visualize the predicted protein–ligand interactions, various tools were used including PyMOL (PyMOL | pymol.org), Protein Plus, and PLIP (Adasme et al., 2021). These tools allowed for a detailed analysis of the complex interactions between the ligands and the MurB enzyme. The combined prediction from these tools was used to examine the potential interactions between the ligands and the MurB enzyme, to identify compounds with the greatest potential for antibacterial activity.

### 2.4 MD simulation

A subset of shortlisted ligand-MurB protein complexes was subjected to MDS to further evaluate their potential as antibacterial agents. The complexes were selected based on their docking score and ADMET analysis. To enable significant conformational changes during MDS, the complexes were produced in each direction of the 10 Å X 10 Å X 10 Å buffer of the gradient box. The TIP4P transferable intermolecular potential was used to introduce water molecules into the system.

The MDS was performed using the Desmond version 4.4 module of Schrodinger’s Maestro 10.4 (Bowers et al., 2006). Before the MDS, energy minimization was performed in 3,000 steps using the steepest descent technique, followed by the conjugate gradient approach in 5,000 steps with a threshold energy of 120 Kcal/mol. During the MDS, constant pressure was maintained using anisotropic diagonal position scaling on a 0.002 ps time step interval. The system was subjected to a 20 ps NPT reassembly at a target pressure of 1 Atm and a slight increase in temperature from 100 K to 330 K. The Lennard-Jones cut-off value and the Berendsen algorithm were set to 0.2 constant and 9 Å, respectively. The SHAKE ideal constraints were applied to all chemical bonds, including those involving hydrogen atoms (Jorgensen and Tirado-Rives, 1988). The minimized structure’s Root Mean Square Deviation (RMSD) was determined by comparing it to the initial structure at 0 ns. This measurement assessed the average variation in atom displacement within a specific frame relative to the initial frame. The RMSD value was computed for every frame throughout the trajectory by using the following equation:

$$\mathrm{RMS}D_{X}=\sqrt{\frac{1}{N}\sum_{i=1}^{N}{\left(r_{i}^{\prime }\left(t_{X}\right)-r_{i}\left(t_{\mathrm{ref}}\right)\right)}^{2}}$$

The system density was kept close to 1 g/cm^3^, and all computations were performed using default settings. The OPLS_2005 force field was used for all calculations. Each complex was subjected to MDS for 100 ns intervals using the same parameters. All simulations were performed in triplicate to ensure the reproducibility of the results. The trajectories obtained from the simulations were analyzed using various tools to assess the stability and conformational changes of the complexes over time. The results of the simulations were evaluated in conjunction with the docking scores and ADMET analysis to identify the most promising ligand-MurB protein complexes for further evaluation as antibacterial agents.

## 3 Results

In the quest for novel therapeutic agents against Mtb, a virtual screening approach followed by MDS was adopted in this study to identify potential inhibitors for the essential MurB enzyme. Based on its significance in Mtb cell biosynthesis, absence in the human body, and documented literature, MurB was selected as the target for this study. A comprehensive screening of compounds from diverse databases was carried out using a Structure-based approach, to identify promising lead compounds for further investigations. Our rigorous methodology enabled the identification of a subset of compounds with a high binding affinity that was subjected to MDS.

### 3.1 MurB screening and docking analysis

**Fig. 3:**
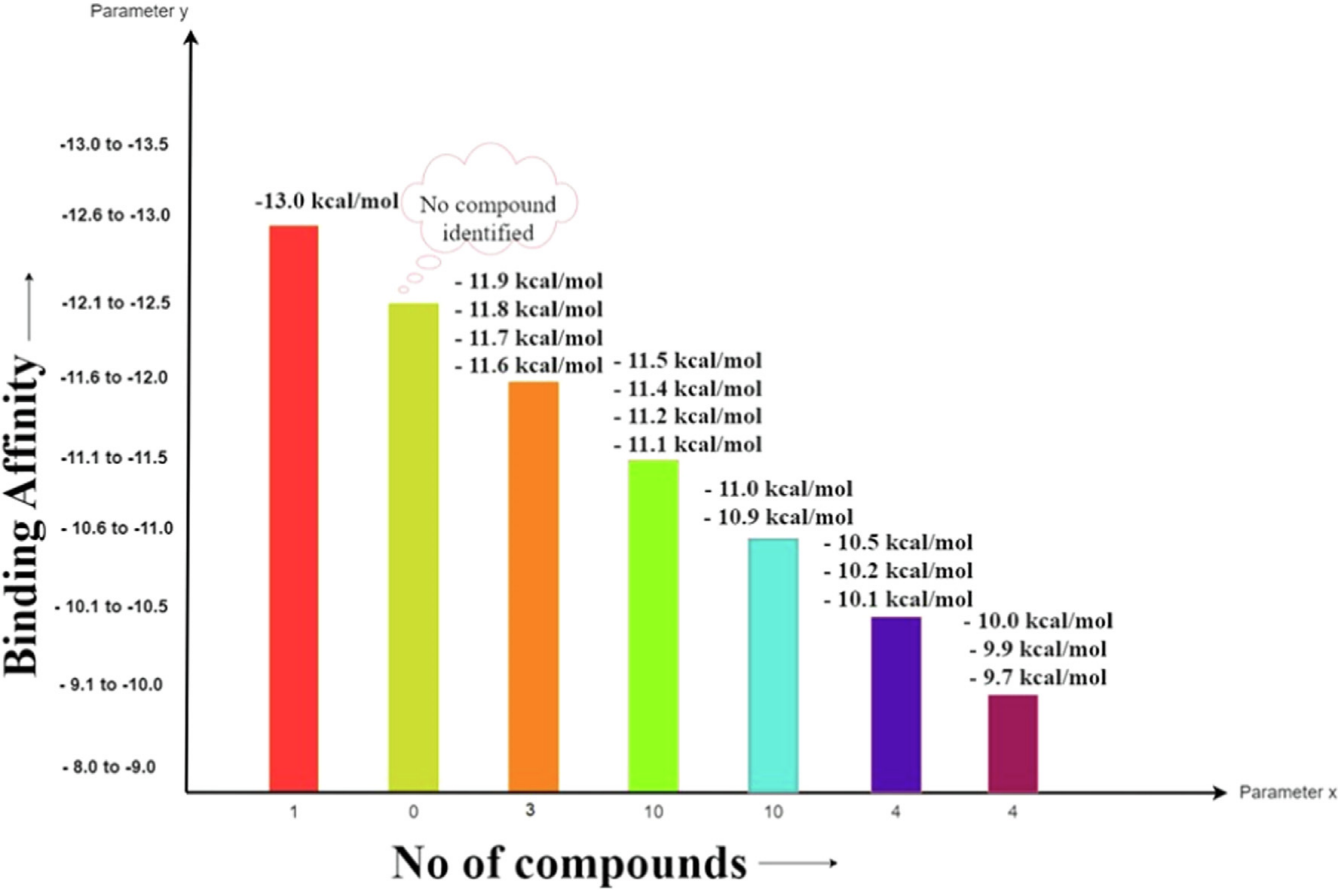
The calculated free binding energies of the top thirty-two hits interacting with MurB have been determined and are reported herein.

Virtual screening has emerged as a powerful tool in modern drug design, enabling the rapid identification of potential drug candidates with a high affinity for target proteins or nucleic acids. Virtual screening was employed to screen vast chemical databases for their potential biological activity against the MurB enzyme. Through the screening approach, thirty-two potential inhibitors against the MurB enzymes were identified and are shown in Fig. 3.

The figure (Fig. 3) presented displays the results of a screening assay to identify compounds with high binding affinity against the target MurB enzyme. The number of compounds identified showed on X-axis and maximum binding affinity on the Y-axis. Notably, one molecule displayed the highest binding affinity of −13.0 Kcal/mol, while the lowest binding affinity identifies was −9.70 Kcal/mol. The binding affinity and inhibition constant values of each compound and other relevant properties are shown in Table 1.

**Table 1:** A comprehensive evaluation of drug-likeness properties of top thirty-two hits against target MurB.

S. No.	Compound ID	Binding Affinity	Inhibition constant (KI)	Molecular Weight	LogP	H-Bond Acceptors	H-Bond	Drug-likeness Score
							Donors	
1	CSID1438694	−11.1	6.87 nM	417.14	5.96	3	1	−0.26
2	CSID2166135	−11	8.15 nM	428.52	5.94	5	2	−0.53
3	CSID1655442	−10.9	9.94 nM	488.55	7.29	5	1	−0.33
4	CSID2154128	−10.5	19.46 nM	448.95	6.28	5	2	−0.16
5	CSID2165834	−10.2	32.80 nM	414.19	4.52	3	2	−0.17
6	CSID2156566	−10.2	33.62 nM	420.88	4.14	5	2	0.1
7	CSID2140363	−10.1	34.00 nM	397.46	5.13	4	1	0
8	CSID2156621	−10	44.61 nM	502.6	5.53	5	2	0.21
9	CSID3866834	−9.9	54.31 nM	469.79	5.7	3	2	0.66
10	CSID2158441	−9.7	68.21 nM	498.95	5.58	4	2	1.25
11	CSID2156999	−9.7	75.09 nM	386.44	3.59	5	2	−0.2
12	DB15688	−13	55.06 pM	638.37	3.53	8	3	1.3
13	DB12983	−11.8	172.62 pM	514.17	7.42	6	2	−1.06
14	DB14773	−11.5	909.21 pM	478.13	4.68	5	2	0.39
15	DB06229	−11.4	182.65 pM	420.2	4.01	5	0	1.05
16	DB12424	−11.2	153.07 pM	557.22	4.27	5	3	1.11
17	DB15396	−11.1	591.56 pM	532.22	4.64	5	1	1
18	DB15401	−11.1	5.35 nM	579.1	2.13	7	1	0.73
19	DB03461	−11	49.86 nM	743.08	−5.74	20	10	0.66
20	DB11852	−11	440.33 pM	517.11	5.97	4	0	0.08
21	DB08901	−11	19.59 pM	532.22	4.66	5	1	1.06
22	ZINC003975327	−11.9	12.34 nM	582.51	5.76	16	0	−0.99
23	ZINC254071113	−11.6	5.21 nM	775.94	6.05	12	3	0.99
24	ZINC084726167	−11.5	557.57 pM	606.74	4.73	7	0	0.17
25	ZINC008215434	−11.3	6.89 nM	785.55	−2.42	23	6	0.77
26	ZINC095539256	−11.1	4.35 nM	777.88	1.91	13	7	1.29
27	ZINC003934128	−11	711.53 pM	680.76	9.86	6	6	−0.13
28	ZINC004215770	−11	5.93 nM	653.63	1.72	13	5	0.61
29	ZINC003780340	−11	19.28 nM	504.45	5.08	8	4	−1.01
30	ZINC001893112	−10.9	18.00 nM	452.48	4.21	6	2	1.1
31	ZINC003978083	−10.9	56.90 nM	609.74	6.7	6	3	1.11
32	ZINC011616153	−9.5	159.32 nM	612.63	3.62	11	4	1.11

Further analysis of the top thirty-two hits is provided, with a detailed description of their respective binding energies. These results provide valuable insight into exploring binding affinity as a critical parameter in identifying potential inhibitors against target enzymes. Our findings suggest these compounds have the potential to be further studied and optimized as potential inhibitors.

### 3.2 ADME and Toxicity analysis

In the pursuit of discovering novel therapeutics, identifying compounds with desirable physicochemical and pharmacokinetic properties is a crucial step toward their success as potential therapeutics. To this end, a comprehensive screening approach utilizing cutting-edge tools such as ADME lab 2.0 (Xiong et al., 2021), pkCSM (Pires et al., 2015), and molsoft L.L.C (https://molsoft.com/mprop/) was employed to identify compounds that possess the necessary attributes for drug candidacy.

To assess the suitability of the identified compounds for further identification, a rigorous evaluation of their physicochemical properties was conducted. This included an analysis of key parameters such as MW, lipophilicity, HBD, HBA, and partition coefficient (LogP), among others (Table 1).

In addition, the aqueous solubility, PBP, HIA, BBB, and tumorigenicity of the compounds were also assessed, as these factors can significantly impact the pharmacokinetic profile of drug candidates (Table 2). These evaluations were performed using established methods and criteria, to identify compounds with favorable drug-like properties and a high potential for success in clinical development.

**Table 2:** ADME and Toxicity analysis of top thirty-two Hits against MurB.

S. No.	Compound ID	PPB (%)	BBB	HIA	Aqueous	Ames	Hepato-	Max. Tolerated dose
					Solubility (moles/L)	Toxicity	Toxicity	
1	CSID1438694	100.62	−0.04	95.18	−5.55	Yes	Yes	0.56
2	CSID2166135	97.47	0.14	92.73	−4.79	Yes	Yes	0.654
3	CSID 1,655,442	97.36	−0.56	89.63	−4.66	Yes	Yes	0.465
4	CSID2154128	93.78	0.1	93.16	−5	No	Yes	−0.551
5	CSID2165834	93.7	−0.08	87.68	−2.89	Yes	No	0.438
6	CSID2156566	94.15	3.99	90.89	−4.7	No	Yes	−0.152
7	CSID2140363	97.05	0	96.81	−6.03	Yes	Yes	0.003
8	CSID2156621	96.56	−0.27	81.73	−2.89	Yes	No	0.438
9	CSID3866834	99.7	4.37	87.12	−5.22	No	Yes	−0.032
10	CSID2158441	95.75	0.101	89.66	−5.46	No	No	0.181
11	CSID2156999	90.29	3.95	92.55	−4.58	No	Yes	−0.169
12	DB15688	81.32	−0.72	87.05	−4	Yes	No	0.438
13	DB12983	95.91	−0.87	78.53	−6.67	Yes	No	0.438
14	DB14773	99.15	−0.23	83.69	−4.81	Yes	No	0.438
15	DB06229	95.56	−0.13	88.71	−4.02	Yes	No	0.438
16	DB12424	93.08	−0.32	80.94	−4.31	Yes	No	0.438
17	DB15396	93.51	0.295	87.5	−4.13	Yes	No	0.438
18	DB15401	95.37	0.62	86.37	−2.7	Yes	No	0.438
19	DB03461	11.77	−5.17	0	−1.15	No	No	0.438
20	DB11852	97.51	0.24	83.96	−6.16	Yes	No	0.438
21	DB08901	93.43	0.39	87.5	−4.22	Yes	No	0.438
22	ZINC003975327	84.03	−2.7	100	−2.89	No	Yes	0.436
23	ZINC254071113	96.9	−2.15	61.57	−3.4	No	Yes	0.52
24	ZINC084726167	83.57	−1.2	92.64	−4.57	No	Yes	−0.214
25	ZINC008215434	67.03	−3.28	22.49	−2.89	No	No	0.274
26	ZINC095539256	89.47	−1.85	50.32	−2.9	No	No	0.335
27	ZINC003934128	98.93	−1.44	100	−2.89	No	No	0.434
28	ZINC004215770	73.46	−1.75	67.67	−3.14	Yes	No	0.176
29	ZINC003780340	77.53	−0.39	100	−2.89	No	No	0.438
30	ZINC003978083	51.37	−2.89	91.33	−2.9	Yes	Yes	0.142
31	ZINC011616153	85.38	−2.058	61.41	−3.46	No	Yes	0.957
32	ZINC001893112	96.67	3.37	92.69	−4.28	No	Yes	0.346

The molecular weight of the compounds ranged from 386.44 to 785.55 g/mol, and the lipophilicity (LogP) ranged from −2.42 to 9.86. In general, for small-drug-like molecules, LogP values can range from about −3 to 6, with most falling within the range of −2 to 4. while the range of the water solubility (LogS) lies from −6.67 to −1.15 mol/L. For most drug-like compounds, LogS values are usually about −5 to 2. Compounds with LogS values below −5 are generally considered to be poorly soluble in water, while LogS values above 2 are typically considered to be highly soluble. To gain deeper insight into the stability and complex interactions between the selected compounds and the target protein, MDS was carried out further.

These findings highlight the meticulous and comprehensive approach taken in evaluating the identified compounds, with a strong focus on key parameters critical to drug discovery and development. This approach is essential in the pursuit of effective treatments for tuberculosis and other diseases caused by bacterial infections and contributes to ongoing efforts to improve global health outcomes.

Using this rigorous screening criteria, a total of thirty-two compounds were identified as potential candidates. Further refinement based on the maximum lower range of binding energies between −11.0 and −13.0 Kcal/mol, resulted in the selection of seven compounds that exhibited exceptional binding properties. These seven compounds were subsequently subjected to MDS to evaluate the stability of their interactions.

### 3.3 Compounds interactions with protein residues

The top seven hits with the binding affinity ranging from −11.0 to −13.0 Kcal/mol were selected for further analysis. Specifically, the interacting residues of the MurB enzyme with these seven compounds were examined to gain insight into their potential mechanism of action and binding pocket. All selected compounds fit well within the target MurB enzyme’s cavity and their postures and interactions were analyzed (Figure S1). Of note, residue Ile 127 was found to be a frequent residue in the pocket where inhibitors against MurB bind, indicating its involvement in the binding of many other molecules.

Detailed analysis of selected compounds revealed distinct hydrophobic and hydrogen bond interactions with specific residues of the target enzyme. For instance, the compounds from ChemSpider with CSID1438694 (Fig. S1A) showed nine hydrophobic interactions and two hydrogen bond interactions, while the compound from DrugBank with DBID12983 (Fig. S1C) interacted with five hydrophobic residues and three hydrogen bond interactions with one π-stacking and one π-cation interaction. The second compound CSID2166135 (Fig. S1B) was found to interact with several specific amino acid residues of the target MurB enzyme. Specifically, it showed interactions with five hydrophobic residues, including threonine at position 26, isoleucine at 127, proline at 128, alanine at 141, and leucine at position 245. In addition, it was found to interact with four amino acid residues via hydrogen bonds, specifically serine at position 70, arginine at 238, serine at 257, and glutamic acid at position 361.

Compound DBID15688 depicted in Fig. S1D manifests an extensive network of molecule interactions comprising thirteen contacts, among which six involve hydrophobic interactions and seven engage in hydrogen bonding. The hydrophobic contacts consist of Ile 127 (two contacts), Val 139, Ala 141, Tyr 175, and Tyr 210. The hydrogen bonding interactions are established with Arg 176, Tyr 210, Ser 257, Asn 261, Glu 302, Ala 325, and Glu 361. This comprehensive interaction profile delineates the intricate interplay of hydrophobic and hydrogen bonding forces governing the binding of DBID15688 to its target, thereby illuminating its potential therapeutic applications.

The compound from Zinc Database, ZINC003975327 (Fig. S1E), interacted with three hydrophobic residues with Tyr 175 (two contacts), and Val 263 and two hydrogen bond interactions with Tyr at position 175 and Thr at 177. The compound ZINC084726167 (Fig. S1F) interacted with three hydrophobic residues and six hydrogen bond interactions. The identified hydrophobic interaction involves Tyr 210, Tyr 287, and Ala 296, while the hydrogen bond interactions are mediated by Tyr 175, Arg 176, Ser 257, Asn 261, and His 324. Additionally, a salt bridge interaction is formed between Glu 361 and the proteins binding partner. Notably, some residues were common among the compounds, suggesting a common binding pocket for these inhibitors. For example, residues Tyr 210 and Tyr 175 were found to have interactions in two compounds, including those with compounds ID DB15688 (Fig. S1D), and ZINC084726167 (Fig. S1F) further supporting the notion of a shared binding pocket.

ZINC254071113 (Fig. S1G) establishes a network of eight molecules interactions with their target enzyme, featuring three hydrophobic and four hydrogen bond interactions with one π-stacking. Specifically, the hydrophobic contacts involve Tyr 210, Pro 283, and Pro 287, while the hydrogen bonding contacts are established with Asn 261, Val 284, His 286, and Gly 298. These results offer a valuable framework for the design of novel MurB inhibitors with enhanced potency and selectivity.

### 3.4 MDS of MurB with ligands

**Fig. 4:**
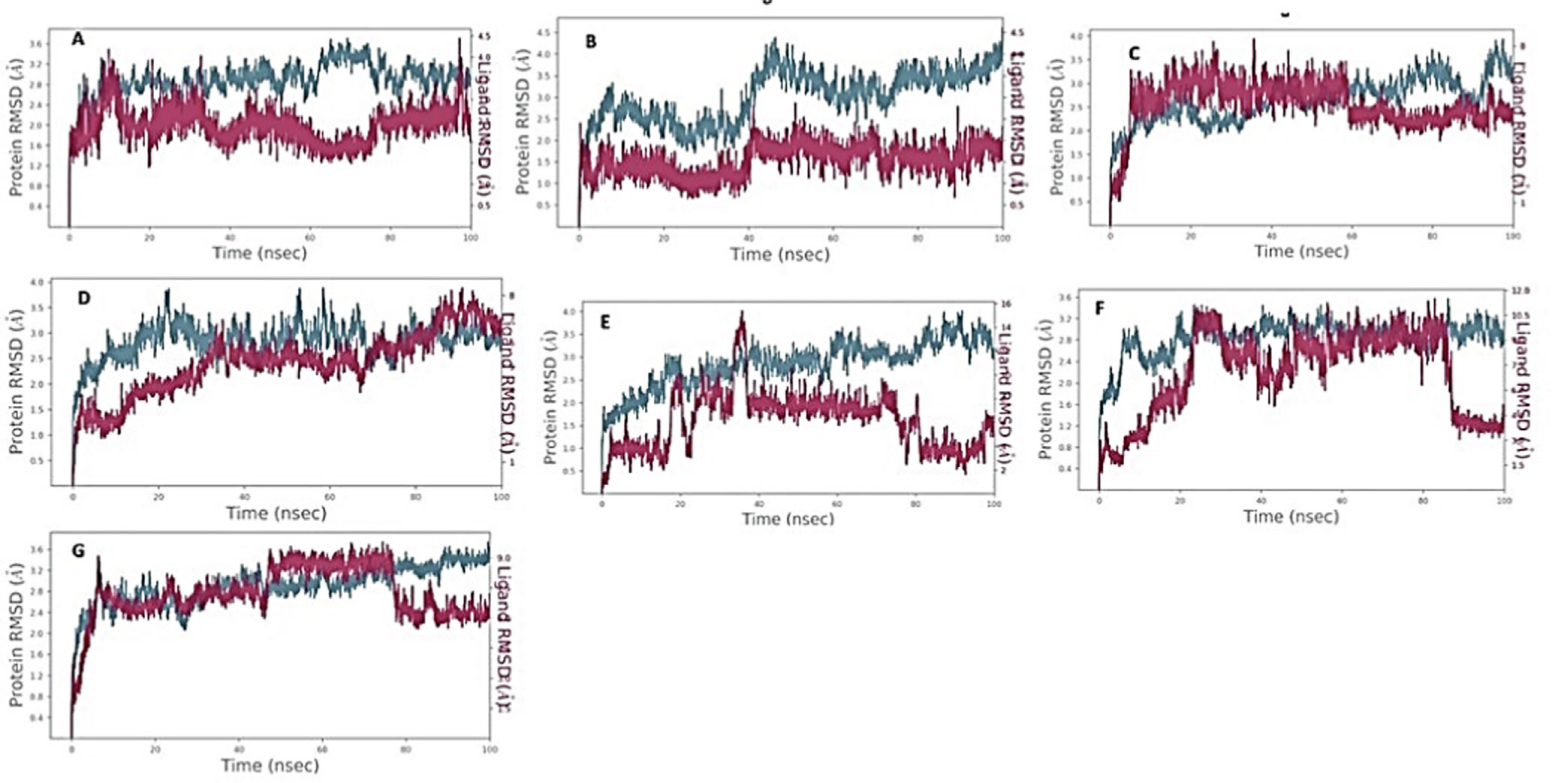
RMSD between MurB of *M. tuberculosis* and selected ligands from various libraries. The ligands shown are A-CSID1438694, B-CSID2166135, C-DB12983, D-DB15688, E-ZINC003975327, F-ZINC084726167, G-ZINC254071113.

The results of the MD trajectory analysis of seven ligands, selected based on their high binding affinity during molecular docking, were examined to determine their potential as inhibitors of MurB protein in tuberculosis drug development. The analysis revealed that four of the seven ligands formed stable complexes with MurB, indicating strong protein–ligand intermolecular interactions (Fig. 4). In particular, CSID1438694 (Fig. 4A) complex demonstrated weak stability and binding within the initial 15 ns, with slight diffusion of the ligand observed between 15 and 20 ns with a maximum deviation of 3.6 Å. A synchronous fluctuation between the protein and ligand was observed in the 20–30 ns timeframe. Although the ligand diffused away from the MurB protein between 40 and 80 ns, it showed weak binding affinity and an RMSD of the protein at 3.2 Angstrom, and the ligand fluctuated sharply at 3.6 Å in the last time frame (80–100 ns). Overall, CSID1438694 exhibited weak binding with MurB protein.

Ligand CSID2166135 (Fig. 4B) did not demonstrate promiscuous binding with MurB protein, showing diffusion away from the protein’s binding site during the 100 ns timeframe, with only slightly weak binding observed at 40 ns. Ligand DB12983 (Fig. 4C) exhibited strong binding with MurB protein during the 10–60 ns timeframe, but later diffused away, with RMSD ranging between 2.2 Å and 3.8 Å, and showed more aberrant fluctuations than the protein during the 20–50 ns timeframe. The second compound from the drug Bank DB15688 (Fig. 4D) exhibits an attractive interaction between the receptor and the ligand. The ligand shows diffusion behavior during the initial 0–35 ns of the simulation, but subsequently, it forms consistent and stable binding interactions with the receptor for the remainder of the simulation times. These findings suggest that DB15688 has the potential to be an effective inhibitor for the receptor of interest. Ligand ZINC003975327 (Fig. 4E) did not exhibit consistent interactions with the target protein, as per RMSD values ranging from 0.5 Å to 3.9 Å for the ligand. Ligand ZINC084726167 (Fig. 4F) undergoes initial diffusion behavior during the initial 0–20 ns of the simulation, followed by a period of consistent and stable binding interaction with the receptor between 20 and 80 ns. Once the ligand establishes a stable binding interaction with the receptor, it forms a complex that can maintain its stability for a prolonged period. However, after 80 ns, the ligand shows a rapid and pronounced dissociation from the receptor.

Ligand ZINC254071113 (Fig. 4G) diffused away from the target after 80 ns timestep, however, a resonance between the alpha carbon atom of the protein backbone and the atomic coordination of the ligand was observed between 10 and 80 ns. Therefore, ligands DB12983 (Fig. 4C), DB15688 (Fig. 4D), ZINC084726167 (Fig. 4F), and ZINC254081113 (Fig. 4G) exhibited stable binding with the MurB protein, with RMSD values ranging between 2.1 Å and 3.6 Å. These findings demonstrate the stability of protein and four out of seven molecules that made the shortlist after MD analysis. The identified compounds exhibit promising inhibition of Mtb and may have a unique mode of action. They could be used as a starting point for chemical modification in medicinal chemistry to create a higher-affinity scaffold with improved inhibitory action.

Our docking and MDS analysis revealed a set of ligands, namely DB12983, DB15688, ZINC084726167, and ZINC254071113, as inhibitors of the MurB enzyme in MTB. Our study revealed that these compounds exhibited consistent and stable interactions with the MurB, displaying very good binding affinity. Our findings indicate that these compounds possess the potential to act as potent inhibitions of Mtb by interacting with the MurB enzyme. Table 3 provides detailed information on the interacting residues of the MurB-ligand complexes, including information on their structure characteristics, hydrophobic and hydrogen bond interactions, and more. Our analysis demonstrates that these four hits remained stable within the active site of UDP-N-acetylenolpyruvoylglucosamine reductase in *M. tuberculosis* and exhibited consistent interactions throughout the simulation.

**Table 3:** Top four hits identified Against Target MurB enzyme and their interacting residues.

Hit ID	Compound ID and Structure	G-Score (Kcal/mol)	H-Bond	Hydrophobic Interactions
Hit 1		−11.8	Tyr 210 Asn 261 Lys 294	Glu 145 Asn 261 Tyr 287 Pro 288 Ala 296
Hit 2		−13.0	Arg 176 Tyr 210 Ser 257 Asn 261 Glu 302 Ala 325 Glu 361	Ile 127 Ile 127 Val 139 Ala 141 Tyr 175 Tyr 210
Hit 3		−11.5	Tyr 175 Arg 176 Arg 176 Ser 257 Asn 261 His 324	Tyr 210 Tyr 287 Ala 296
Hit 4		−11.6	Asn 261 Val 284 His 286 Gly 298	Tyr210 Pro283 Pro287

The table (Table 3) likely summarizes the results of molecular docking or MD study that aimed to identify potential ligands that could bind to the MurB enzymes. The table may also list the specific residues in the MurB enzymes that were found to be involved in ligand–protein interactions. This information can provide insight into the mechanism of binding and help in designing more effective inhibitors.

## 4 Discussion

The emergence of drug resistance has become a major concern in the fight against infectious diseases, necessitating the search for new compounds with unique mechanisms of action. The development of novel molecules across different classes entails a series of intricate processes, which include the discovery of new compounds with distinct mechanisms of action, the identification of potential inhibitors, and the chemical modification of existing drugs. These processes are crucial in the pursuit of effective and safe therapeutic agents for various ailments. Several approaches have been extensively utilized to discover potential inhibitors, such as high throughput screening, whole-cell-based screening, and combinatorial synthetic chemicals. These techniques have facilitated the identification of a diverse array of Mur enzyme inhibitors sourced from different organisms and a plethora of antitubercular scaffolds (Hrast et al., 2013, Hrast et al., 2014). Currently, these inhibitors are being assessed at varying stages of clinical trials, suggesting their promising therapeutic properties (Loerger et al., 2013).

Bedaquiline represents a successful outcome of using target-based high-throughput screening to identify an antitubercular drug that hinders the MTB’s ATP synthase enzyme (Kundu et al., 2016). The availability of small-molecule libraries and the progress in computational techniques have presented more opportunities for discovering novel chemical scaffolds that target specific proteins. However, despite these advancements, the effectiveness of TB drug discovery research remains hindered by the absence of experimental validation of in silico hits and the challenge of translating in-vitro activity into mycobactericidal activity and vice versa (Eniyan et al., 2020).

In recent times, Mur enzymes found in Mtb have gained significant attention as a potential drug target owing to their indispensable role in the survival of the pathogen (Kouidmi et al., 2014, Jukič et al., 2019, Yang et al., 2006). Readers are directed to a comprehensive review by Hrast and colleagues which highlights an array of broad-spectrum chemical inhibitors that effectively target bacterial Mur ligases (Hrast et al., 2014). This review delivers into the mechanistic underpinnings of these inhibitors and the structural features that facilitate their binding to MurB. Several inhibitors targeting MurB have been documented in the literature (Bronson et al., 2003, Francisco et al., 2004, Kutterer et al., 2005). Additionally, the significance of MurB as a target in Mtb is supported by scientific studies. For example, a study by Eniyan and colleagues, provided insight into the structure and function of MurB, emphasizing its significance in the peptidoglycan biosynthesis pathway (Eniyan et al., 2018). Another research study by Rani and colleagues, aimed to identify potential drugs that could inhibit MurB enzymes, suggesting MurB is a potential target of MTB (Rani et al., 2020). A research study by Kumar and colleagues contributes to our understanding of the structure and function of the MurB enzyme and provides valuable insights for future drug discovery efforts targeting peptidoglycan biosynthesis (Kumar et al., 2011). Furthermore, other research studies by Bronson and colleagues have highlighted the essentiality of MurB in Mtb, finding presents opportunities for the development of novel antibacterial agents that can effectively target the MurB enzyme and potentially address antibiotic-resistant bacterial infections (Bronson et al., 2003). According to the studies conducted by Kumar and colleagues, the compound under investigation serves as a substrate for the MurB, which function as a reductase and exerts its catalytic activity on the substrate. Notably targeting the MurB enzyme presents an opportunity for selective inhibition, as it is not present in the Human system. This characteristic renders the MurB a promising candidate for identifying inhibitors with potential therapeutic implications (Kumar et al., 2020).

To identify inhibitors that are specific to the MurB enzyme, various compound repositories were screened in this study. The MurB enzyme was selected for the screening process as crystal structures of this enzyme in Mtb have been previously solved. We used 30,417 compounds from three different databases to screen for MurB inhibitors. From the initial screening, the top thirty-two compounds were selected based on their binding affinity, drug-likeness, and ADMET properties. To assess the stability and interactions of the compounds with the MurB enzymes, MDS was performed. Among the screened compounds, DB12983, DB15688, ZINC084726167, and ZINC2540741113 emerged as the most favorable candidates based on their strong binding affinity, stable conformations, and robust interactions with the key residues. The binding affinity of these compounds, as indicated by their docking score of −11.8 Kcal/mol, −13.0 Kcal/mol, −11.5 Kcal/mol, and −11.6 Kcal/mol, respectively, suggests their potential as potent inhibitors of MurB*.*

Analysis of the PLIP revealed that these compounds interacted strongly with key residues, namely Tyr 175, Asn 261, Tyr 210, Arg 176, and Ser 257. Tyr 175 were found to be common in multiple ligands including DB12983, DB15688, and ZINC084726167 indicating their essential role in the binding of these compounds. Ser 257 is found to have common interaction in DB15688 and ZINC084726167. Additionally, Asn 261 and Tyr 210 were also found to be common in DB12983, DBID15688, ZINC254071113, and ZINC084726167, suggesting their critical role in the binding of these inhibitors to MurB*.* The results of this study revealed that four potential compounds interact with previously reported residues in the active site MurB of *M. tuberculosis.* These residues have been previously shown to play a critical role in the enzyme’s activity and are considered important targets for inhibition by Daffé and Marrakchi (Daffé and Marrakchi, 2019). The fact that the potential compounds identified in this study interact with these key residues suggests that they may have the potential to inhibit MurB activity and serves as promising lead compounds for further optimization.

These compounds can be used as a basis for further chemical alterations and refinements aimed at enhancing their inhibitory effects and creating scaffolds with greater affinity. The MDS provided valuable insight into the stability and interactions of the compounds with the MurB enzyme, which could aid in the development of more effective inhibitors of MTB.

This investigation presents a successful approach that combines a Structural-based screening with MDS to efficiently identify potential inhibitors for further development. Our results demonstrate the potential of this methodology for high-throughput screening of larger compound libraries, providing a valuable tool for drug discovery research.

## 5 Conclusion

In conclusion, our study has identified four promising compounds, DB12983, DB15688, ZINC084726167, and ZINC254071113, that can effectively inhibit the MurB enzyme, presenting a potential strategy for inhibiting the initial stage of PG biosynthesis in *M. tuberculosis*. These compounds have demonstrated excellent binding and stable conformation with the MurB enzyme, and have the potential to serve as effective antimycobacterial agents, including drug-resistant strains. Further experimental validation of these compounds as potential inhibitors is warranted, and may pave the way for the development of novel antimicrobial therapies for tuberculosis. The current study represents a significant breakthrough in the field of tuberculosis research, as we are the first to report the efficacy of these compounds against drug-resistance *Mycobacterium tuberculosis*. This finding is particularly noteworthy because drug resistance is a major barrier to the treatment of tuberculosis, which has caused significant challenges in global health. Our study provides a potential solution to this problem by identifying new compounds that can effectively inhibit the MurB enzyme, an essential component of tuberculosis cell wall synthesis. By targeting this enzyme, our compounds have the potential to overcome the resistance mechanism of tuberculosis and serve as an effective therapy for drug-resistant strains. Additionally, the study may have employed innovative approaches such as computational approach, high-throughput screening, or structure-based drug design to identify these potential inhibitors. These promising results open up new avenues for the development of novel antimicrobial therapies and have significant implications for the future treatment of tuberculosis.

## Funding

The authors are grateful to the Researchers Supporting Project number (RSP2023R360), King Saud University, Riyadh, Saudi Arabia.

## CRediT authorship contribution statement

**Ankit Verma:** Writing – review & editing, Writing – original draft, Visualization, Validation, Software, Resources, Methodology, Investigation, Formal analysis, Data curation, Conceptualization. **Vijay Kumar:** Writing – review & editing, Writing – original draft, Visualization, Validation, Supervision, Software, Resources, Methodology, Formal analysis, Data curation, Conceptualization. **Bindu Naik:** Writing – review & editing, Writing – original draft, Validation, Investigation, Formal analysis, Data curation, Conceptualization. **Javed Masood Khan:** Writing – review & editing, Writing – original draft, Resources. **Pallavi Singh:** Writing – review & editing, Methodology. **Per Erik Joakim Saris:** Writing – review & editing, Writing – original draft. **Sanjay Gupta:** Writing – original draft, Resources.

## Declaration of Competing Interest

The authors declare that they have no known competing financial interests or personal relationships that could have appeared to influence the work reported in this paper.

## Appendix A Supplementary data

Supplementary data 1 Figure S1. cell wall of Mtb’s MurB, where the docked compounds are in contact with antagonistic residues. The hydrogen bond interactions are denoted by yellow dotted lines, A- MurB-CSID1438694; B- MurB-CSID2166135; C- MurB-DB12983; D-MurB-DB15688; E- MurB-ZINC003975327; F- MurB-ZINC084726167; G-MurB-ZINC254071113.
