# Supplementary figures and images for: Screening and molecular dynamics simulation of compounds inhibiting MurB enzyme of drug-resistant Mycobacterium tuberculosis: An in-silico approach
Source: Saudi J Biol Sci. 2023 Jul 4;30(8):103730. doi: 10.1016/j.sjbs.2023.103730 (PMC10362793; doi:10.1016/j.sjbs.2023.103730)

## A. MurB-CSID1438694

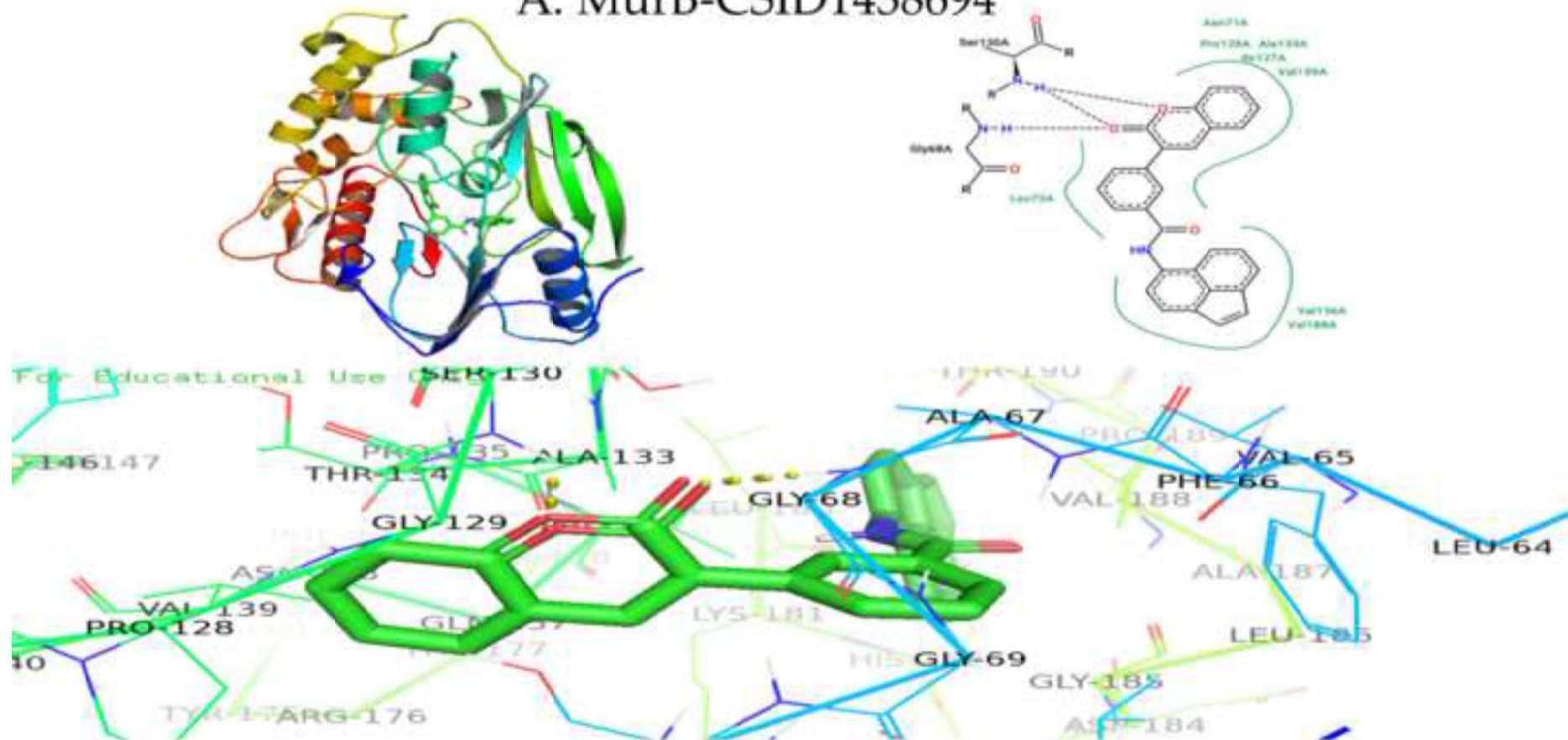

## B. MurB- CSID2166135

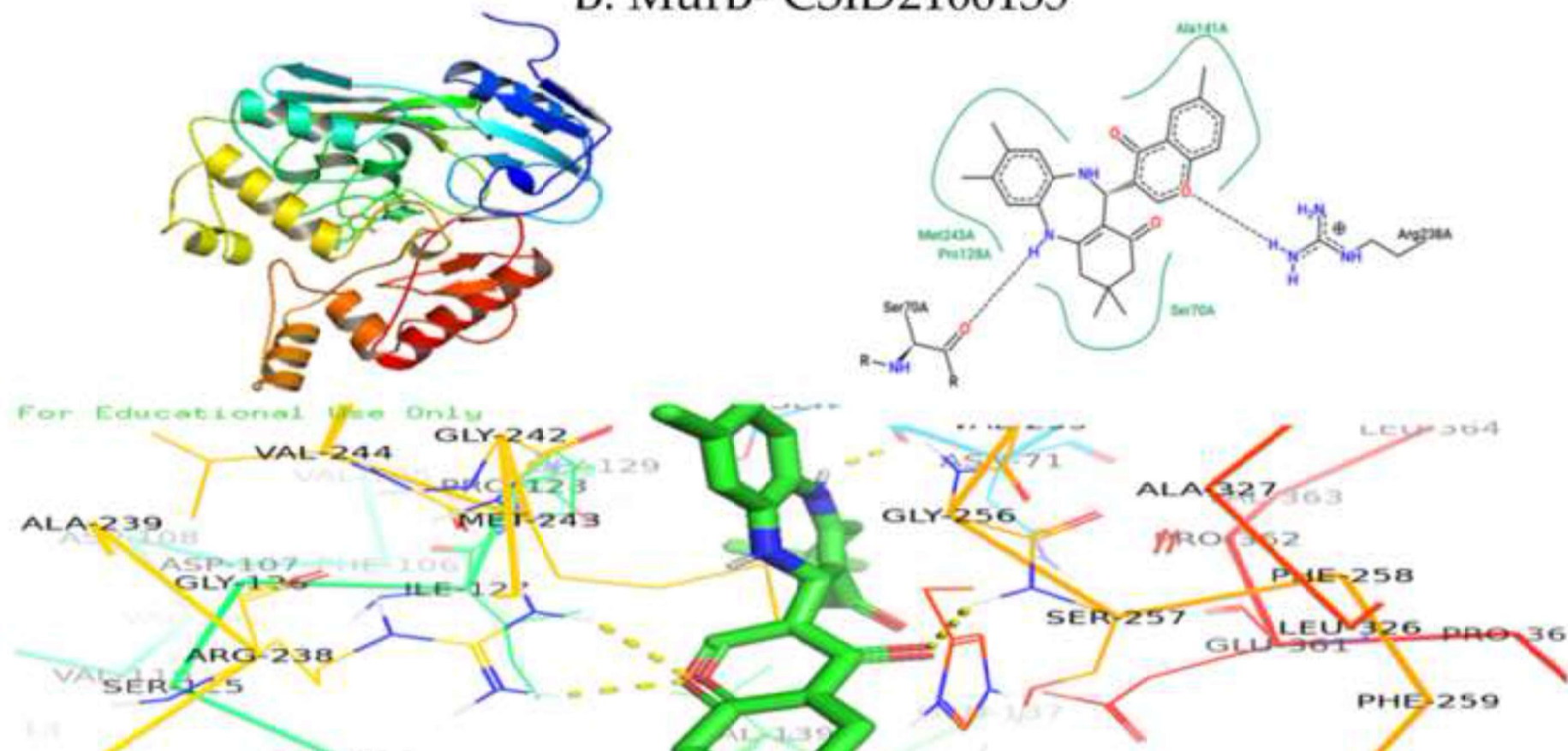

## C. MurB-DB12983

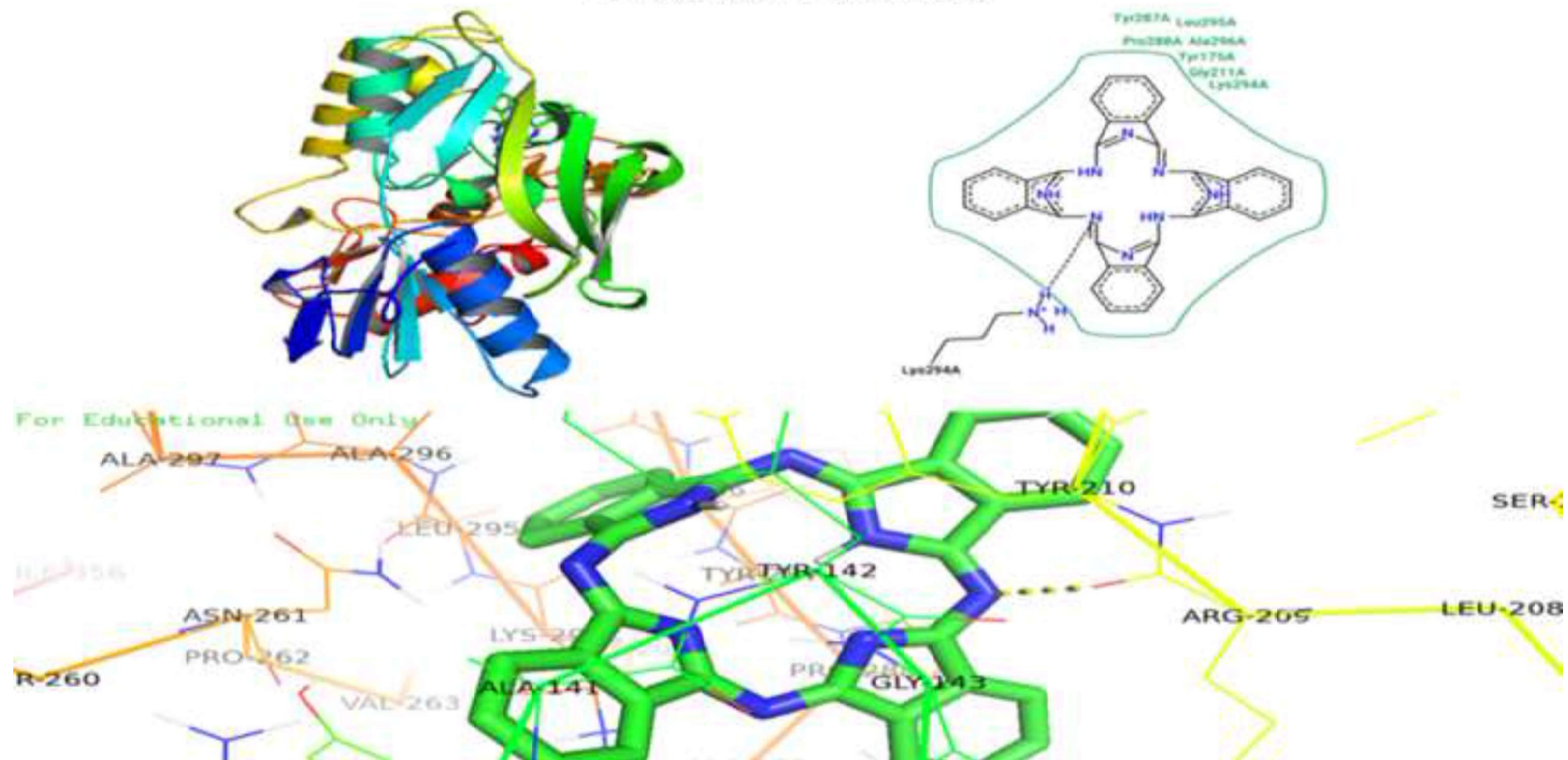

## D. MurB- DB15688

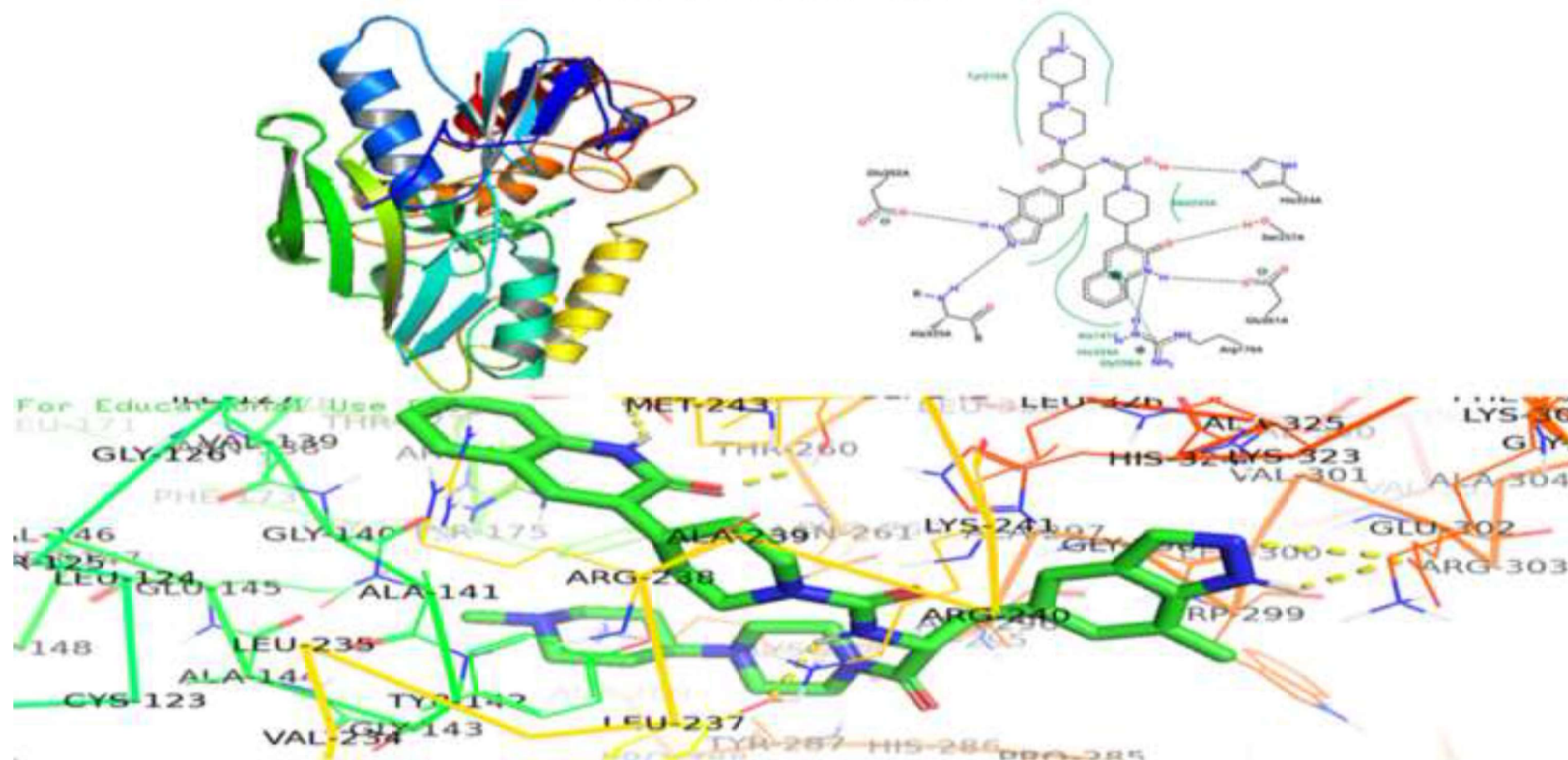

## E. MurB-ZINC000003975327

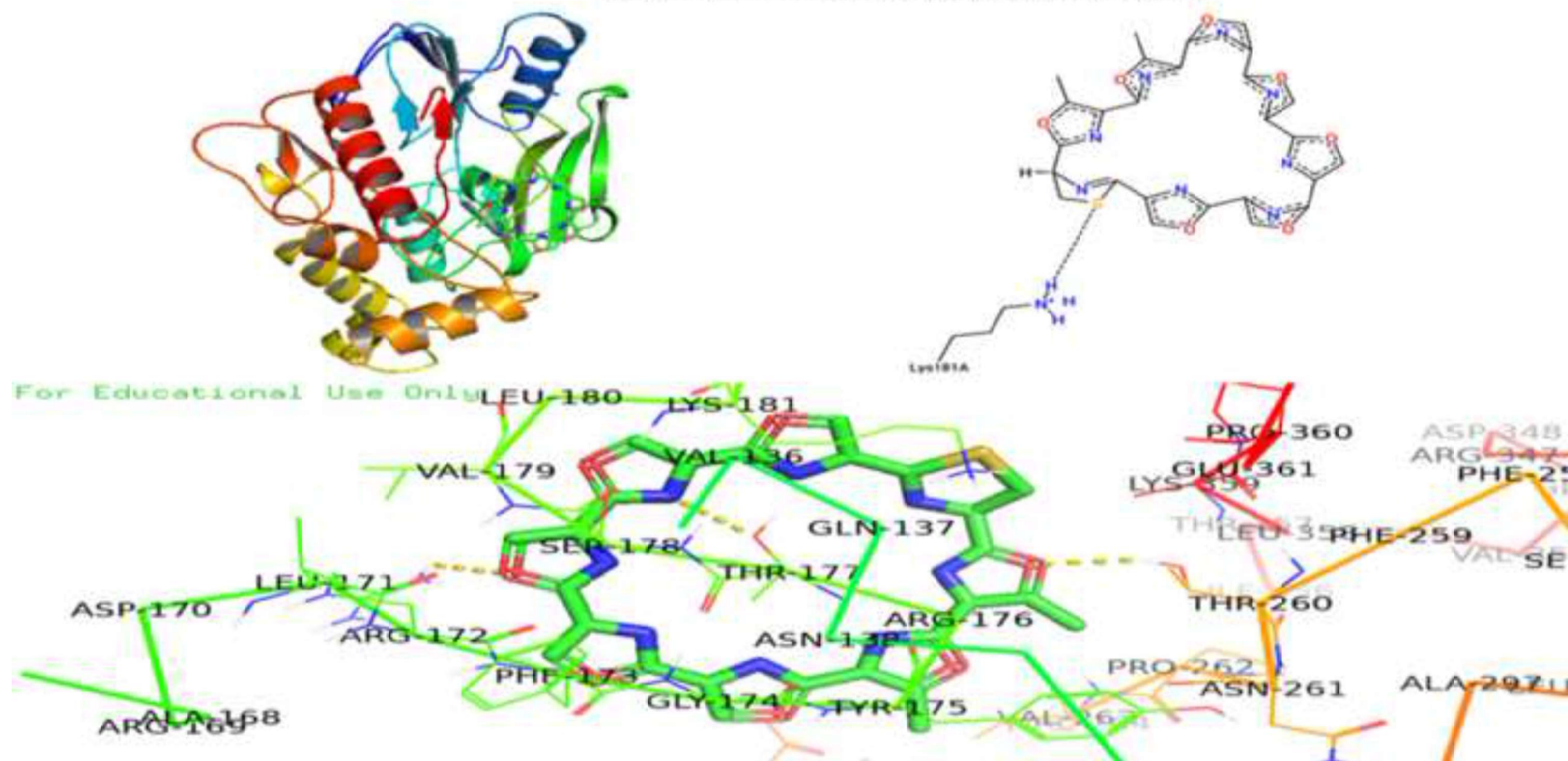

## F. MurB- ZINC000084726167

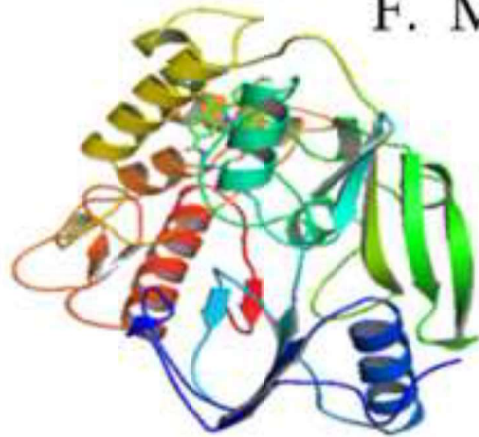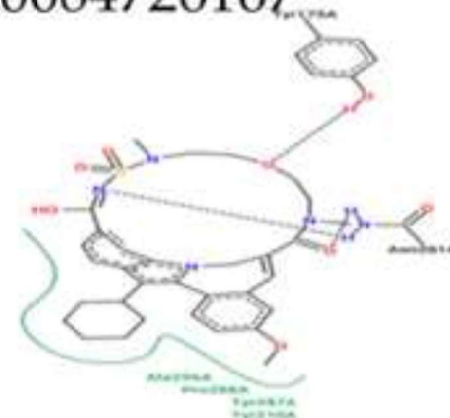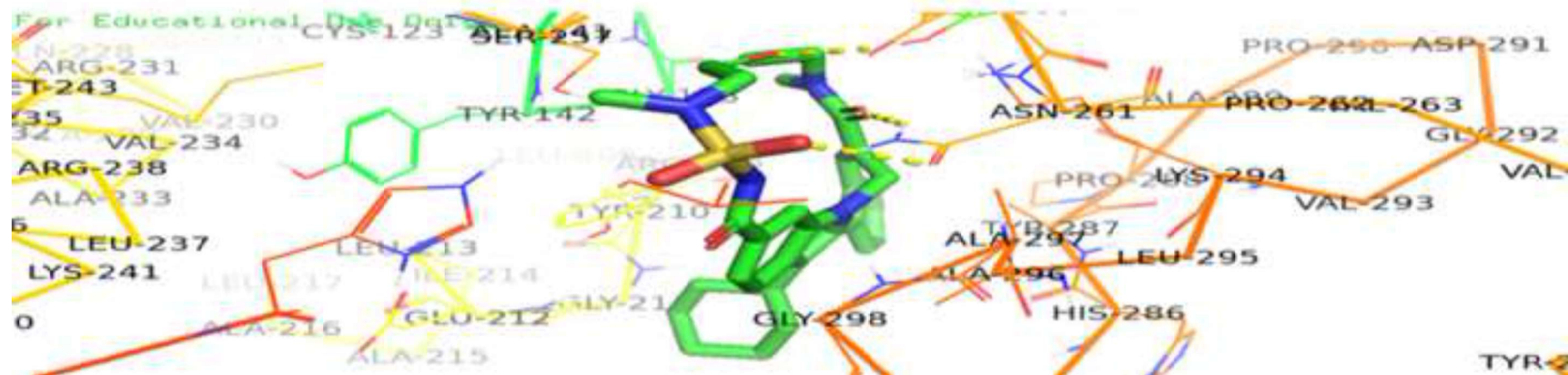

## G. MurB-ZINC000254071113

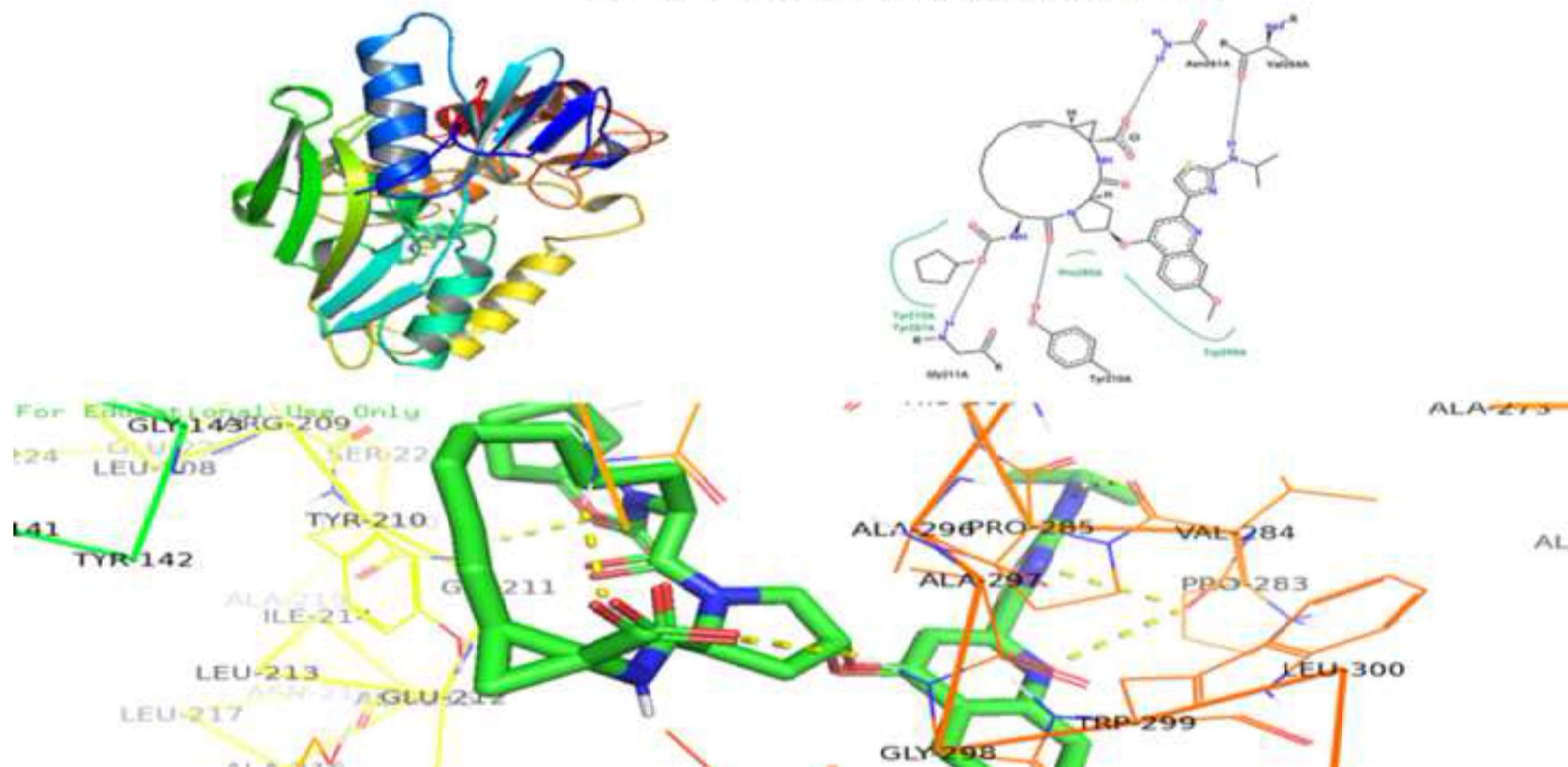

Supplement: Supplementary data 1 — Figure S1. cell wall of Mtb’s MurB, where the docked compounds are in contact with antagonistic residues. The hydrogen bond interactions are denoted by yellow dotted lines, A- MurB-CSID1438694; B- MurB-CSID2166135; C- MurB-DB12983; D-MurB-DB15688; E- MurB-ZINC003975327; F- MurB-ZINC084726167; G-MurB-ZINC254071113. [file mmc1.pdf]
